# Real-Time Segmentation of Tactile Paving and Zebra Crossings for Visually Impaired Assistance Using Embedded Visual Sensors

**DOI:** 10.3390/s26030770

**Published:** 2026-01-23

**Authors:** Yiqiang Jiang, Shicheng Yan, Jianyang Liu

**Affiliations:** 1 School of Mechanical and Electrical Engineering, Yibin University, Yibin 644000, China; 2018123001@yibinu.edu.cn; 2 School of Mechanical Engineering, Southwest Jiaotong University, Chengdu 610031, China; yanchengysc@gmail.com

**Keywords:** semantic segmentation, lightweight model, embedded platform, visual impairment assistance

## Abstract

This study aims to address the safety and mobility challenges faced by visually impaired individuals. To this end, a lightweight, high-precision semantic segmentation network is proposed for scenes containing tactile paving and zebra crossings. The network is successfully deployed on an intelligent guide robot equipped with a high-definition camera and a Huawei Atlas 310 embedded computing platform. To enhance both real-time performance and segmentation accuracy on resource-constrained devices, an improved G-GhostNet backbone is designed for feature extraction. Specifically, it is combined with a depthwise separable convolution-based Coordinate Attention module and a redesigned Atrous Spatial Pyramid Pooling (ASPP) module to capture multi-scale contextual features. A dedicated decoder efficiently fuses multi-level features to refine segmentation of tactile paving and zebra crossings. Experimental results demonstrate that the proposed model achieves mPA of 97% and 93%, mIoU of 94% and 86% for tactile paving and zebra crossing segmentation, respectively, with an inference speed of 59.2 fps. These results significantly outperform several mainstream semantic segmentation networks, validating the effectiveness and practical value of the proposed method in embedded systems for visually impaired travel assistance.

## 1. Introduction

According to relevant research [1,2], as of 2020, approximately 1.1 billion people worldwide were living with varying degrees of visual impairment, including about 43 million who were completely blind, 295 million with moderate to severe visual impairment, and 258 million with mild visual impairment. With the continued growth of the global population and the acceleration of population aging, the number of people with complete blindness is projected to increase to 115 million by 2050. Because visually impaired individuals have difficulty obtaining complete information about their surroundings (such as paths, obstacles, and traffic signals), their frequency of outdoor activities typically decreases, leading to a significant decline in their quality of life [3].

Although many countries have improved travel conditions for visually impaired individuals by installing infrastructure such as tactile paving and accessible pathways, the effectiveness of these measures is often hampered by incomplete coverage, obstructions (e.g., parked vehicles, street vendors), and inadequate maintenance. This is primarily because essential environmental information (such as temporary obstacles and dynamic traffic) cannot be fully perceived, thereby increasing safety concerns during outdoor activities [4]. At present, visually impaired individuals primarily rely on assistive tools such as white canes or guide dogs when traveling outdoors. A white cane allows users to assess the environment ahead by tapping the ground. Although it is simple to use, it offers limited perception of rapidly approaching obstacles. Guide dogs provide more reliable navigation; however, their training and maintenance costs are high, and the application process is cumbersome.

In recent years, with the rapid development of the Internet of Things (IoT) and sensor technologies, research on intelligent assistive devices for healthcare has achieved significant progress. Intelligent navigation assistance for the visually impaired has become one of the major research directions, with existing work including smart canes [5,6,7], robotic guide dogs [8,9], and wearable smart navigation devices [10,11]. These systems typically rely on multiple sensors (such as ultrasonic sensors, LiDAR, infrared/thermal imaging devices, and RGB cameras) to acquire environmental information and assist visually impaired individuals in walking safely. The accuracy and robustness of environmental perception directly determine device performance; therefore, perception algorithms constitute the core component of such systems. Moreover, because guidance devices are constrained by size and computational resources, perception algorithms must balance accuracy with real-time performance and low computational overhead [12].

Among various environmental sensors, vision sensors are widely used in smart guidance systems because their sensing mechanism closely resembles human visual perception. Vision-based tactile paving and zebra crossing segmentation techniques have advanced rapidly, and their high-accuracy segmentation results can effectively enhance the independent mobility of visually impaired individuals while reducing safety risks [13]. Existing segmentation methods can be broadly classified into two categories: methods based on traditional image feature extraction and methods based on deep learning. Traditional methods typically utilize low-level features such as color and texture to achieve object segmentation. Although these methods generally require low computational resources, they exhibit poor robustness to illumination changes, occlusions, and complex backgrounds, resulting in substantial performance fluctuations in real-world applications [14].

In recent years, the rapid development of deep learning has led to its widespread application in image segmentation tasks. Compared with traditional handcrafted feature extraction methods, deep learning significantly enhances feature representation capability and segmentation accuracy through end-to-end learning. Zhang et al. [15] proposed a tactile paving segmentation method with multi-scale feature extraction by integrating a group convolution strategy with a group receptive field block to enable cross-channel information interaction. Although this method produces rich feature representations, its large number of parameters and high computational complexity limit deployment on resource-constrained devices. To address this issue, Wan et al. [16] designed a multimodal semantic segmentation model, Sigma, based on the Mamba architecture, which integrates thermal images, depth images, and RGB images, achieving strong segmentation performance even under low-light or overexposed conditions. Li et al. [17] proposed a tactile paving segmentation approach based on a diffusion model and introduced a voting mechanism to reduce the impact of initial random noise, thereby improving overall segmentation accuracy. Tokita et al. [18] further focused on the problem of irregular tactile paving installation and developed a segmentation model based on the DeepLabV3+ framework that can distinguish between warning-type and guiding-type tactile paving. Although existing tactile paving semantic segmentation models have achieved strong accuracy, the limited computational resources of guiding devices for the visually impaired pose challenges for deploying these high-precision models on embedded platforms. Therefore, lightweight model design has attracted increasing research attention.

Research on the lightweighting of deep learning networks can generally be divided into two categories [19,20,21]. The first focuses on network architecture design, aiming to construct models with simpler structures and fewer parameters. The second focuses on post-training model compression, which reduces the number of parameters through techniques such as pruning, quantization, and distillation to achieve lightweight deployment. For example, Yang et al. [22] proposed a lightweight road-surface segmentation model based on DeepLabV3+, in which the parameter count is significantly reduced, thereby greatly lowering the computational demand of terminal devices. Niu et al. [23] proposed a fast tactile paving segmentation model based on re-parameterization. In this model, the down-sampling module re-parameterizes a multi-branch training network into a single-branch inference network, ensuring faster inference speed and lower computational resource consumption. Shi et al. [24] addressed the issue of resource constraints and proposed a context-aware lightweight segmentation network. The designed partial-channel transformation (PCT) strategy not only enriches extracted contextual features and reduces overfitting risk but also enables accurate pixel-level predictions with fewer parameters and lower computational cost. In addition, Zhang et al. [25] integrated relation-based and feature-based knowledge distillation to develop a more accurate and lightweight real-time road-surface segmentation model, which more effectively satisfies the real-time requirements of embedded systems.

In summary, this study aims to deploy a high-accuracy semantic segmentation model on embedded platforms, enhancing inference speed while maintaining segmentation accuracy. To this end, we designed and implemented an intelligent guide-assist vehicle system and deployed the proposed lightweight segmentation approach on its embedded platform to achieve real-time segmentation of tactile paving and zebra crossings, thereby assisting visually impaired individuals in travelling safely. In addition, regarding data, we collected and created an image dataset of tactile paving and zebra crossings under various lighting, environmental, and geometric conditions. Simultaneously, we merged it with other public datasets for training and testing, enhancing the robustness of the model. Detailed information about the dataset created and utilized in this paper will be elaborated in Section 3.1. The proposed lightweight network can efficiently and accurately perform tactile paving and zebra crossing segmentation without requiring complex pre-processing, such as image-enhancement strategies.

**Figure 1:**
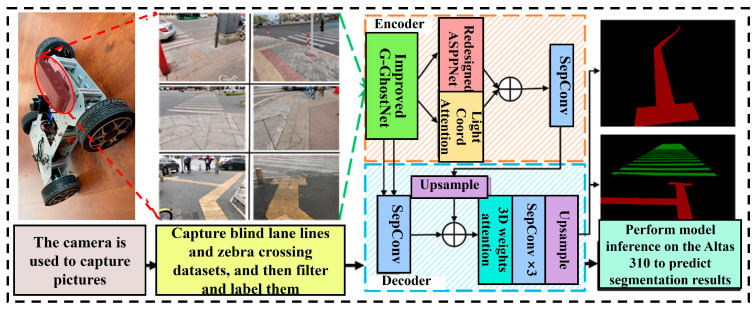
System architecture of the proposed embedded platform.

Figure 1 illustrates the workflow of the proposed system, which consists of four main components: (1) a high-definition camera used to capture tactile paving and zebra crossing images; (2) construction of a tactile paving and zebra crossing dataset; (3) a fast and lightweight semantic segmentation method based on an improved DeepLabV3+ architecture, in which the network is optimised through the integration of attention mechanisms and depthwise separable convolutions to enhance segmentation accuracy while reducing parameter complexity; (4) deployment of the proposed model on the Atlas 310 embedded processor (Huawei Technologies Co., Ltd., Shenzhen, China), followed by validation on the vehicle platform.

The main contributions of this paper are as follows:

- We designed and implemented an intelligent guide-assist vehicle equipped with a high-definition camera and an Atlas 310 embedded processor, and successfully deployed the proposed lightweight segmentation network on this platform to achieve real-time tactile paving and zebra crossing segmentation.
- We propose an improved G-GhostNet backbone that incorporates depthwise separable convolutions and a Mix operation, significantly enhancing segmentation inference speed.
- We enhanced the spatial pyramid pooling module by integrating an attention mechanism for multi-scale feature extraction, and designed a decoding module tailored to tactile paving and zebra crossing characteristics to improve segmentation accuracy and edge-preservation performance.

## 2. Method

**Figure 2:**
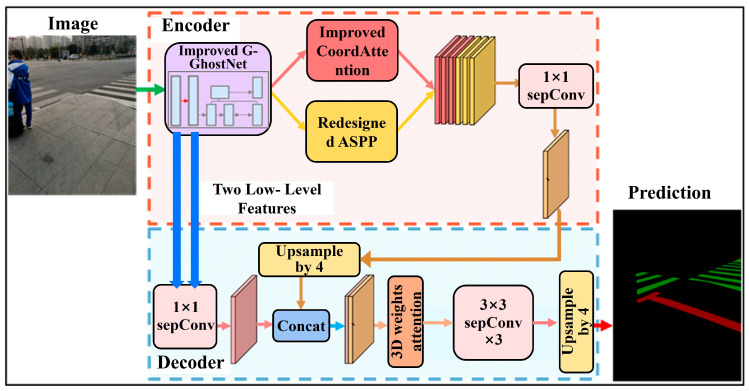
Overall architecture of the proposed semantic segmentation algorithm.

The lightweight semantic segmentation network for tactile paving and zebra crossings developed in this study is illustrated in Figure 2. The network follows an encoder–decoder architecture. In the encoder, an improved G-GhostNet backbone is first employed to extract the initial features from the input image. The resulting low-level features are then passed to a parallel feature enhancement structure composed of an improved Coordinate Attention module and an improved Atrous Spatial Pyramid Pooling (ASPP) module, enabling the extraction of deeper and multi-scale contextual features. The decoder fuses the low-level features from the backbone with the enhanced features and further refines the feature representation through a 3D weights attention mechanism. Finally, three depthwise separable convolutional layers combined with upsampling operations are applied to generate the final segmentation predictions for tactile paving and zebra crossings.

### 2.1. Hardware System Architecture

**Figure 3:**
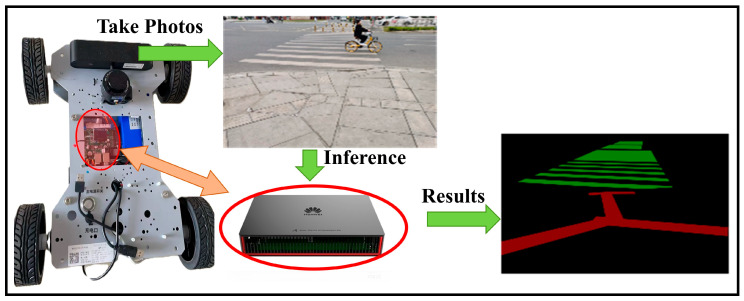
Hardware System Architecture Diagram.

An intelligent guide vehicle system, illustrated in Figure 3, is constructed in this study and consists of a high-definition camera and a Huawei Atlas 310 (Huawei Technologies Co., Ltd., Shenzhen, China) embedded computing unit. To accommodate the computational limitations of the embedded platform, a pruned and quantized semantic segmentation model is deployed on the Atlas 310 for inference. The system first captures real-time images of tactile paving and zebra crossings through the camera and transmits the images to the Atlas 310 embedded processor. The built-in graph compilation engine compiles and optimizes the deployed model, thereby accelerating inference and enabling rapid perception of the surrounding environment. Finally, the recognized tactile paving and zebra crossing information is transmitted to the control module of the intelligent guide vehicle to assist visually impaired individuals in safe navigation.

### 2.2. Encoding Module

The encoder architecture, illustrated in the upper part of Figure 2, consists of three submodules: (1) an improved G-GhostNet module for extracting the initial semantic features; (2) an improved Coordinate Attention module for enhancing channel and spatial attention; and (3) a redesigned ASPP module for capturing multi-scale contextual information.

#### 2.2.1. Improved G-GhostNet Module

**Figure 4:**
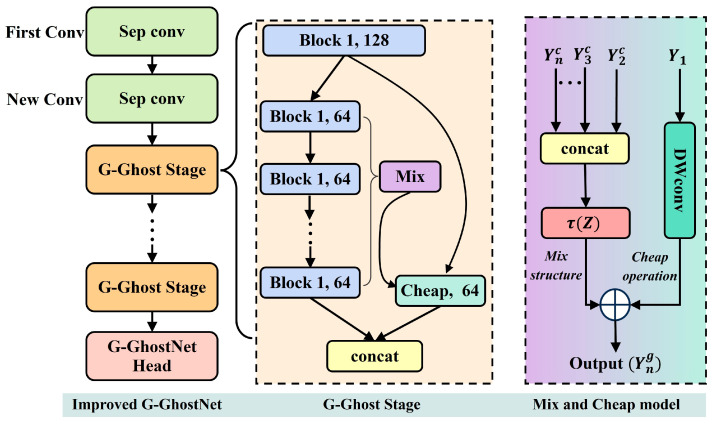
Architecture of the improved G-GhostNet module.

To enhance inference efficiency and feature representation capability while a lightweight architecture, an improved backbone module based on G-GhostNet is designed to serve as the primary feature extractor, as shown in Figure 4. Compared with typical lightweight networks (e.g., the MobileNet family) and traditional backbones (e.g., ResNet), G-GhostNet exhibits superior parallel efficiency on platforms like GPUs and NPUs. Capitalizing on this inherent efficiency, we further optimized its branch structure to better utilize the computational resources of embedded devices.

The improved G-GhostNet module consists of two convolutional layers, multiple G-Ghost Stages, and a G-GhostNet Head. Each G-Ghost Stage contains multiple Block modules and a Mix module, where the Mix module fuses the output features from different Blocks, enabling the extraction of more discriminative features while maintaining low computational cost.

Specifically, a complex feature map $Y_{n}^{c}$ is obtained through a full-size block of size 1 × 128 and multiple half-size blocks of size 1 × 64. Meanwhile, the Mix module collects the operation results of half size blocks and obtains the aggregated feature τ(Z) through global pooling and full connection. Subsequently, Cheap operation is performed on the full-size block operation results to obtain $C(Y_{1})$. Then, τ(Z) and $C(Y_{1})$ are combined to produce the cheap feature $Y_{n}^{g}$. Finally, the complex feature map is fused with the cheap feature obtained by the Mix module to generate the output feature $Y_{n}$ of the improved G-GhostNet backbone. The detailed computation process is presented in Equation (1), where $Y_{1}$ represents the feature map obtained by the full-size block, $\left\{L_{2},L_{3},\cdots ,L_{N}\right\}$ represents a series of half-size convolution blocks, and $Y_{i}^{c}$ represents the output feature maps of the corresponding blocks. Concat and Pooling denote the channel concatenation and pooling operations, respectively, which are used to obtain the aggregated feature τ(Z).

With the incorporation of the Mix module, the improved G-GhostNet enhances its capability to represent complex textures while maintaining low computational overhead.

(1) $$\left\{\begin{array}{l} Y_{n}^{c}=L_{n}(L_{n-1}(\cdots L_{2}(Y_{1})))\\ Z=Concat(Y_{2}^{c},Y_{3}^{c},\cdots ,Y_{N}^{c})\\ \tau (Z)=W*Pooling(Z)+b\\ Y_{n}^{g}=C(Y_{1})+\tau (Z)\\ Y_{n}=\left[Y_{n}^{c},Y_{n}^{g}\right] \end{array}\right.$$

#### 2.2.2. Improved Coordinate Attention Module

**Figure 5:**
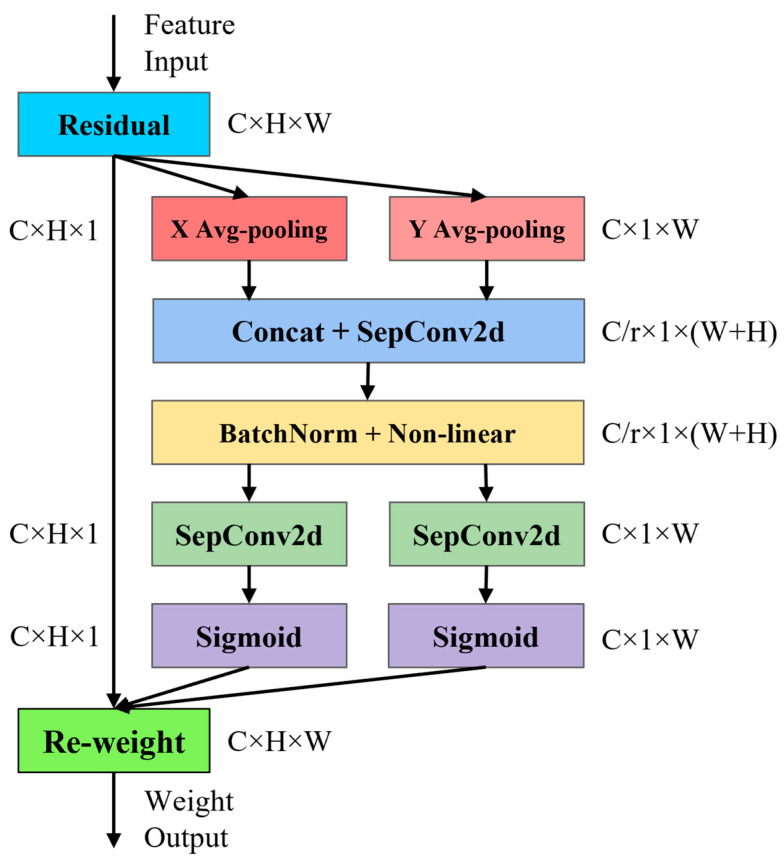
Architecture of the improved coordinate attention module.

Considering the computational constraints of embedded platforms, we replaced the standard convolutions in the original coordinate attention module with depthwise separable convolutions in order to reduce the computational burden, as illustrated in Figure 5. This modification preserved the module’s spatial and positional encoding capabilities while preventing an increase in computational overhead.

First, the input feature map F undergoes average pooling along the X and Y axes separately to obtain feature maps $f_{w}(w)$ and $f_{h}(h)$ in the width and height directions. Subsequently, the feature maps in the width and height directions are concatenated and passed through a depthwise separable convolution with a 1 × 1 kernel to reduce their dimensionality. After normalization, the reduced feature map f is processed using depthwise separable convolutions along the height and width dimensions, generating two attention weights $F_{h}$ and $F_{w}$, which correspond to the original height and width, respectively. Finally, the original feature map is multiplied by $F_{h}$ and $F_{w}$ to obtain the final feature map $F_{finall}$. The specific process can be represented by Equation (2), where $f_{h}(h)$ and $f_{w}(w)$ denote the feature maps obtained after performing pooling along the height and width directions of the feature map, respectively, while H and W represent the height and width of the feature map data. σ and BatchNorm correspond to the Sigmoid activation function and batch normalization operation, respectively, and SepConv denotes depthwise separable convolution.

(2) $$\left\{\begin{array}{l} \begin{array}{cc} f_{h}(h)=\frac{\sum_{0\leq i\leq w}F(h,j)}{W}; & f_{w}(w)=\frac{\sum_{0\leq j\leq H}F(j,w)}{H} \end{array}\\ f=\sigma (BatchNorm(\left[f_{h},f_{w}\right]))\\ \begin{array}{cc} F_{h}=\sigma (SepConv(f^{h})); & F_{w}=\sigma (SepConv(f^{w})) \end{array}\\ F_{finall}=F\times F_{h}\times F_{w} \end{array}\right.$$

#### 2.2.3. Redesigned ASPP Module

**Figure 6:**
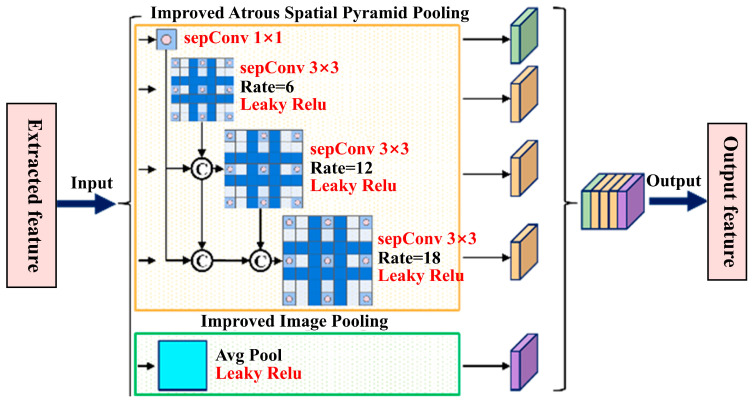
Architecture of the redesigned ASPP module.

To more effectively extract multi-scale features, we redesign the ASPP module based on dilated convolutions, as illustrated in Figure 6. This module employs dilated convolution branches with dilation rates of 1, 6, 12, and 18, along with an average pooling branch, to capture contextual information at different scales from the input feature map.

First, dilated convolutions with dilation rates of 1 and 6 are applied to the input feature map, and their outputs are fused with the input feature map to obtain the first-stage fused features. These fused features are then fed into the dilated convolution layer with a dilation rate of 12. Furthermore, the outputs of the dilated convolutions with dilation rates of 1, 6, and 12 are fused again with the input feature map to obtain the second-stage fused features, which are then used as the input for the dilated convolution with a dilation rate of 18. Finally, the outputs of all dilated convolution layers are concatenated and passed through a 1 × 1 convolution for channel fusion, yielding the final multi-scale fused features.

To further reduce the number of model parameters, we integrate depthwise separable convolutions into the redesigned ASPP module. This integration significantly decreases both parameter count and computational cost while preserving the module’s multi-scale modeling capability.

### 2.3. Decoding Module

To achieve accurate segmentation of tactile paving and zebra crossing under varying weather, color, and occlusion conditions, we designed a decoder module inspired by DeepLabv3+, as illustrated in the lower part of Figure 2. In designing the decoder, we considered that tactile paving and zebra crossing may exhibit highly similar colors and textures, and relying solely on shallow features from the first layer of the improved G-GhostNet module may lead to insufficient semantic information and blurred boundaries. Therefore, all features extracted by the two depthwise separable convolution layers in the improved G-GhostNet module were fed into the decoder, and a 1 × 1 pointwise convolution was applied for channel reduction before fusing them with the backbone output features to reduce computational cost.

**Figure 7:**
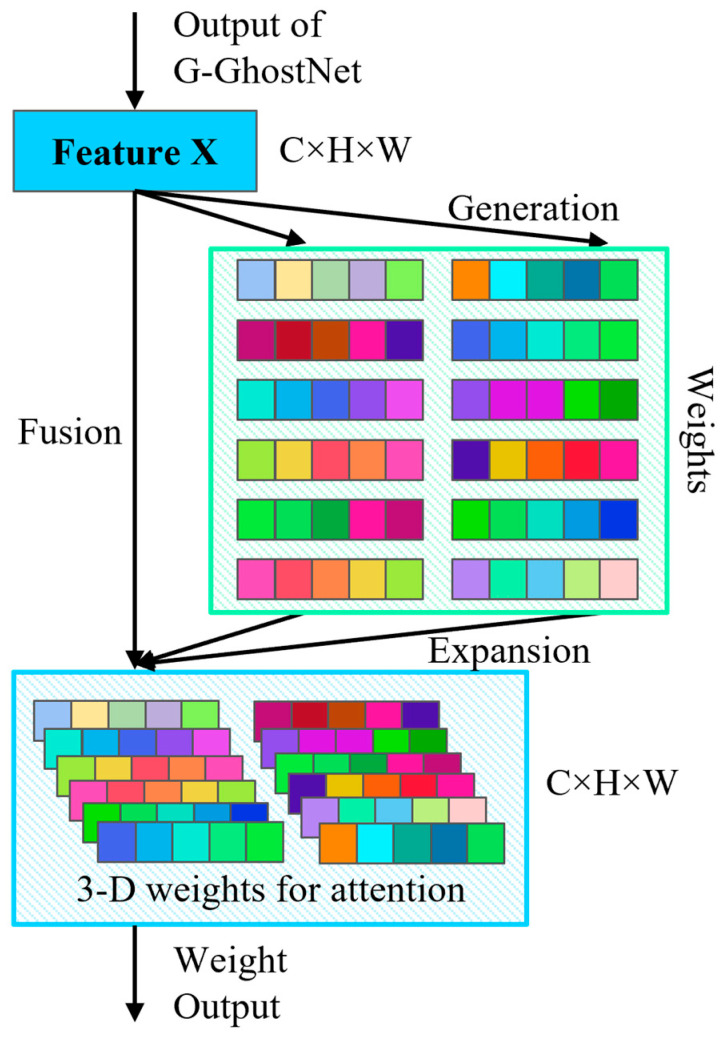
Architecture of the 3D weights attention module.

To further enhance local detail restoration, we designed a lightweight 3D weights attention module, as shown in Figure 7, to fuse channel and spatial information. Unlike other attention mechanisms, the 3D weights attention module adopts a neuron activation mechanism similar to that of the human brain, where an energy function is used to weight the input feature maps to obtain channel and spatial attention weights. These weights are then multiplied with the original input feature map to generate the fused feature representation with both channel and spatial attention. Thus, this module required no additional parameters and incurred a low computational cost, while effectively improving the model’s attention distribution across spatial and channel dimensions. The detailed energy function and the computational flow of the 3D weighted attention module are given in Equations (3) and (4), respectively. Here, F, E, and $F_{3D}$ denote the input feature map, the energy coefficient, and the final 3D weighted feature map, respectively; $\hat{\sigma }$ and $\hat{\mu }$ represent the variance and mean of each channel of the feature map; λ is a constant that prevents division by zero; and ⊙ denotes element-wise multiplication. Finally, the decoder fuses all feature maps using three 3 × 3 depthwise separable convolutions, and a final 1 × 1 convolution was applied to map the number of channels to the number of classes.

(3) $$E=\frac{4({\hat{\sigma }}^{2}+\lambda )}{{\left(F-\hat{\mu }\right)}^{2}+2{\hat{\sigma }}^{2}+2\lambda }$$

(4) $$F_{3D}=sigmoid(\frac{1}{E})⊙F$$

## 3. Experiments

To validate the reliability and robustness of the proposed model, this study conducts comparison experiments and ablation experiments to enable a comprehensive evaluation of the model’s performance. This section presents the experimental environment, dataset construction, evaluation metrics, and the training and testing details

Firstly, all models are implemented using the PyTorch (version 1.7.1) deep learning framework and trained on the same GPU server. The server runs Ubuntu 18.04 and is equipped with an Intel 6× Xeon E5-2678 CPU and an NVIDIA RTX 2080Ti 11 GB GPU. After training, the models were cross-compiled and deployed on the Huawei Atlas 310 embedded platform, which is equipped with eight ARM A55 CPUs @ 1.6 GHz, two DaVinci AI Core NPUs, and 8 GB of general-purpose RAM.

### 3.1. Dataset and Evaluation Metrics

The tactile paving and zebra crossing dataset constructed in this study consists of 407 images and four video sequences. All images have a resolution of 512 × 512 and cover three typical scenarios: tactile paving, zebra crossings, and their combinations. The four video sequences were captured by a high-definition camera mounted on a smart guide vehicle under various weather and scene conditions, all with a resolution of 1024 × 1024, and are used to evaluate the real-time segmentation performance of the model in real driving scenarios. The 407 images were annotated frame by frame and randomly divided into 307 training images, 50 validation images, and 50 testing images. To enhance the model’s generalization ability and make full use of existing data resources, this study merges our self-constructed dataset with public datasets such as tactile paving dataset (TP-Dataset) [15] and Sidewalk Segment Dataset [26] to form the overall data pool for this research. Subsequently, it is divided into training, validation, and test sets, with sizes of 1000, 500, and 500 images, respectively. The specific composition and division of each data source are detailed in Table 1.

The model is evaluated using common semantic segmentation metrics, including mean pixel accuracy (mPA), mean intersection over union (mIoU), overall accuracy (Accuracy), and mean recall (mRecall).

### 3.2. Experimental Details

**Figure 8:**
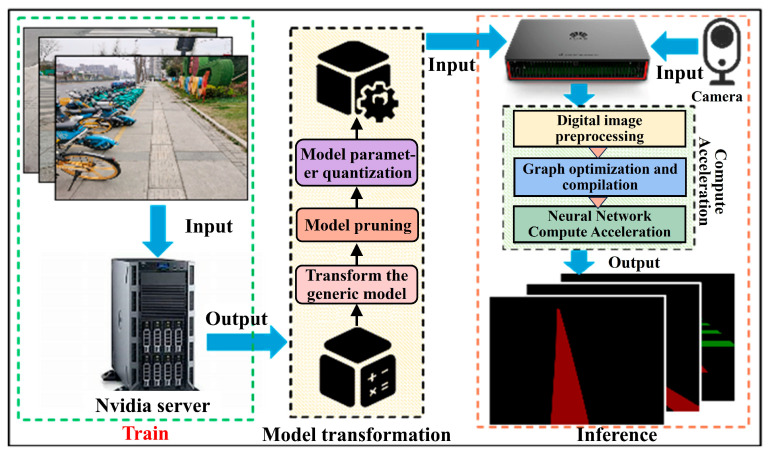
The training and inference pipeline of the proposed method.

The experimental process, as illustrated in Figure 8, consists of two stages: training and inference. Due to the limited training data available, and to simulate scenarios such as camera contamination and insufficient lighting, we performed data augmentation on the training images to improve the model’s generalization ability and reduce the risk of overfitting. Specific augmentation strategies included injecting random noise and reducing image brightness by 0–10%. To ensure experimental fairness to the greatest extent possible, the hyperparameters listed in Table 2 were used for model training.

After training, the model undergoes format conversion, pruning, and quantisation to produce a lightweight inference model suitable for the embedded platform. This process mainly refers to the official manual and guidelines of Huawei Ascend, which will not be repeated here. During inference, the high-definition camera on the guide vehicle captures images in real time and transmits them to the Atlas 310, where the neural network inference is accelerated through the platform’s hardware scheduling mechanism, ultimately producing real-time segmentation results.

### 3.3. Comparative Experimental Analysis

**Figure 9:**
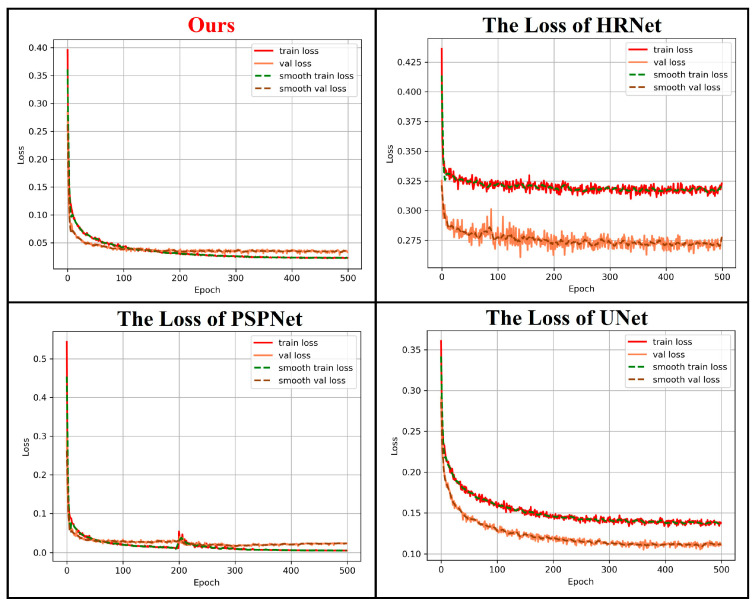
Loss curves of different models during training.

In comparative experiments, our proposed model was compared and analyzed with typical semantic segmentation models HRNet [27], PSPNet [28], and UNet [29]. All models are trained with the same training parameters as described earlier. The training loss curves are shown in Figure 9, from which it can be observed that the convergence trend of ours is consistent with that of the other methods.

**Figure 10:**
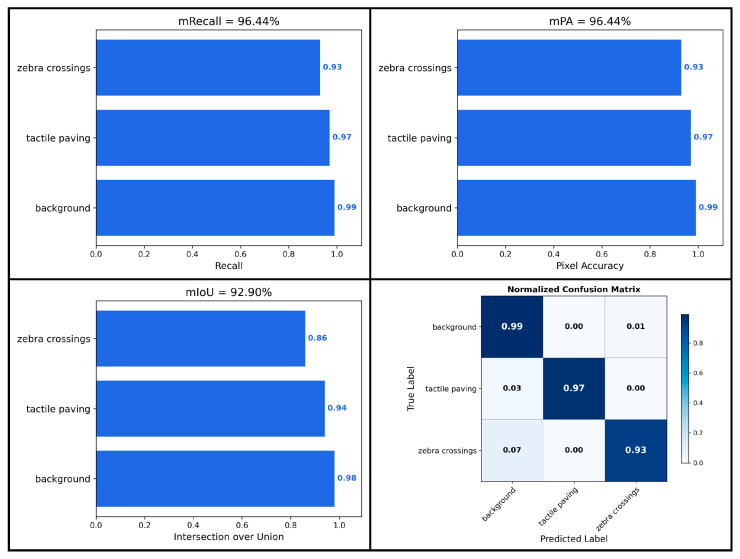
Performance metrics and confusion matrix of the proposed model.

From the performance metrics and confusion matrices shown in Figure 10, it can be observed that our model achieves an mPA of 97% and an mIoU of 94% in tactile paving segmentation, and reaches an mPA of 93% and an mIoU of 86% in zebra crossing segmentation. Meanwhile, Table 3 presents the comparative performance results between ours and other semantic segmentation models. Although PSPNet has clear advantages in terms of parameter count and computational speed, its segmentation accuracy is considerably lower than that of ours. Specifically, its Accuracy, mPA, and mIoU are lower by 3.58%, 7.05%, and 14.38%, respectively. Compared with UNet and HRNet, our model not only possesses a lower parameter count but also demonstrates superior segmentation accuracy. In addition, ours achieves an inference speed of 59.2 fps, which meets the real-time requirements for assisting visually impaired individuals during navigation.

**Figure 11:**
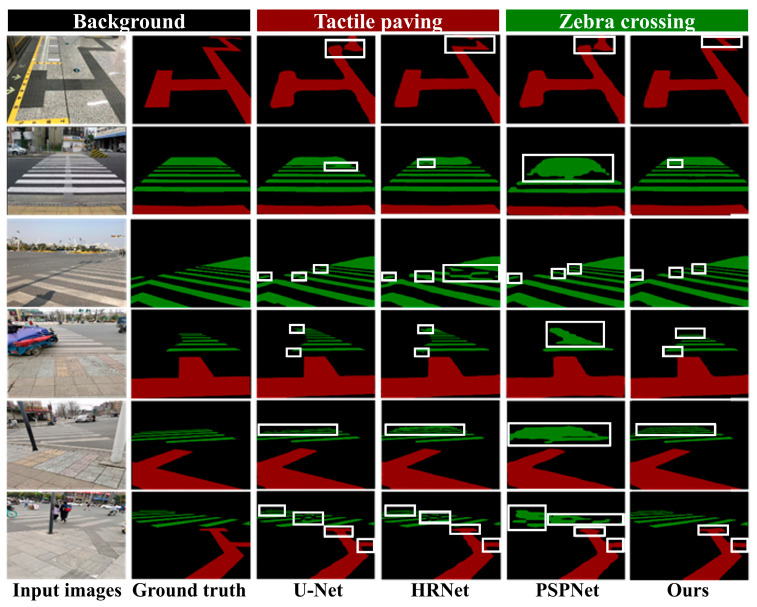
Visualization of segmentation results for different models.

Beyond the quantitative metrics, the visual segmentation results shown in Figure 11 illustrate that our model maintains stable and clear segmentation performance under challenging conditions such as complex illumination, shadows, surface wear, and occlusions, as clearly evidenced by the details highlighted within the white boxes.

To further validate the practical performance of these models in real embedded scenarios, we deployed them on an intelligent guide robot platform and conducted a quantitative evaluation using our video data. The specific results are shown in Table 4, where the symbols ↓ and ↑ respectively indicate a performance decrease or increase on the embedded platform compared to the server. It can be observed that after deployment on the embedded platform, PSPNet, UNet, and HRNet exhibit a noticeable decline across all metrics compared with our model. In particular, the mIoU of HRNet decreases by 3.31% compared to its pre-deployment performance, while our model only drops by 0.34%. Moreover, in terms of inference speed on the embedded device, our model requires only 17.3 ms, which is second only to PSPNet, effectively meeting the real-time requirements of embedded systems.

### 3.4. Ablation Studies

To further verify the effectiveness of the key modules in our model, we conduct a series of ablation experiments, and the results are presented in Table 5.

First, to examine the contribution of the 3D weights attention mechanism introduced in the decoder module, we evaluate its impact independently. When this module is removed, the model’s mIoU decreases by 1.81%. Moreover, across different configurations, removing this module consistently leads to performance degradation, indicating that it enhances feature attention capability during the decoding stage and thereby improves the segmentation results.

Secondly, we evaluated the effectiveness of the parallel feature extraction network. When the parallel structure was replaced with a serial structure, the mIoU exhibited a significant decline, indicating that the parallel feature extraction network plays a critical role in capturing features of different scales and granularities.

Specifically, when the improved coordinate attention module was removed while retaining only the redesigned ASPP module, the model’s mIoU decreased by 6.8%. Conversely, when the redesigned ASPP module was removed and only the improved coordinate attention module was retained, the mIoU dropped to just 53.69%, and the model was nearly unable to correctly identify tactile paving and zebra crossing. These results demonstrate that both the improved coordinate attention module and the redesigned ASPP module, as well as the parallel feature extraction network they form, make decisive contributions to the overall performance of the model.

Finally, the ablation results clearly show that each module in our model is of great importance in enhancing feature extraction capability, strengthening multi-scale information fusion, and improving the segmentation accuracy of tactile paving and zebra crossing. The synergy among these components enables the model to maintain high segmentation performance across various scenarios.

## 4. Discussion and Conclusions

This study proposes a lightweight, high-precision semantic segmentation network designed for tactile paving and zebra crossing scenarios. The network was successfully deployed on an intelligent guide vehicle system, which utilizes a high-definition camera and an Atlas 310 embedded computing platform. In practice, the system achieved excellent performance. First, we collected and constructed a dataset containing tactile paving and zebra crossing, providing a reliable data foundation for model training. Subsequently, the improved G-GhostNet was adopted as the feature extraction network, effectively extracting the basic features of input images while significantly reducing the number of model parameters. To further enhance feature representation, we introduced an attention mechanism integrated with depthwise separable convolutions and redesigned the Atrous Spatial Pyramid Pooling (ASPP) module, achieving extraction and enhancement of multi-scale features. Moreover, we designed a decoding module for efficiently fusing multi-level features, ensuring high-quality segmentation performance in complex road scenarios. Experimental results show that our model significantly improves segmentation accuracy and inference speed while maintaining low computational cost. It outperforms several mainstream networks, thereby validating its effectiveness and practical value for embedded visual perception tasks. For future work, we plan to augment the dataset and refine the model architecture to achieve a better balance between computational efficiency and segmentation accuracy, with the ultimate goal of meeting the stringent requirements of real-time assistive mobility devices.

While the proposed system demonstrates robust performance in standard scenarios, its evaluation under more diverse and challenging conditions, such as low-light and varying weather, remains a focus for future work due to the limitations of the current integrated dataset. Furthermore, subsequent efforts will be directed toward collecting real-world scene data from visually impaired individuals and conducting supervised user-needs analysis, ultimately enabling closed-loop validation of the system’s practicality and safety.

## Figures and Tables

**Table 1:** Composition and partitioning of the datasets used in this study.

	Training Set	Validation Set	Test Set	For Deployment
Our dataset	307	50	50	Video Data (4 sequences)
TP-Dataset	800	200	200	--
Sidewalk Segment Dataset	893	350	350	--
Total	2000	600	600	Video Data (4 sequences)

**Table 2:** Model training parameters.

**Parameters**	Initial Learning Rate	Batch Size	Epoch	Optimizer	Lr Decay
**Value**	0.007	26	500	SGD	cos

**Table 3:** The comparative experiment results.

Methods	PSPNet	UNet	HRNet	Ours
Accuracy	95.02%	97.15%	97.58%	98.60%
mPA	89.39%	95.85%	96.69%	96.44%
mIoU	78.52%	93.67%	92.82%	92.90%
PA _Tactile paving_	92.92%	95.16%	93.77%	97.05%
PA _Zebra crossing_	78.97%	91.93%	89.47%	93.14%
IoU _Tactile paving_	87.53%	93.16%	92.31%	94.34%
IoU _Zebra crossing_	53.80%	85.17%	85.82%	86.01%
Params size	5.34 MB	24.89 MB	29.55 MB	19.51 MB
FPS	75.9	52.1	41.8	59.2

**Table 4:** Performance comparison of different models on the embedded platform.

Methods	PSPNet	UNet	HRNet	Ours
Accuracy	92.92% (↓ 2.21)	96.19% (↓ 0.96)	97.47% (↓ 0.11)	97.15% (↓ 1.45)
mPA	89.80% (↑ 0.41)	92.87% (↓ 2.98)	94.88% (↓ 1.81)	95.01% (↓ 1.43)
mIoU	76.43% (↓ 0.09)	91.06% (↓ 2.61)	89.51% (↓ 3.31)	93.24% (↑ 0.34)
Latency	14.3 ms	21.2 ms	30.6 ms	17.3 ms

**Table 5:** The ablation experiment results.

Improved G-GhostNet	Improved Coord ATT	Redesigned ASPP	3D Weights ATT	mIoU	PA _Tactile Paving_	PA _Zebra Crossing_
✓	--	✓	--	85.21%	86.67%	82.69%
✓	--	✓	✓	86.14%	88.86%	86.43%
✓	✓	--	--	52.06%	53.73%	48.92%
✓	✓	--	✓	53.69%	53.81%	51.07%
✓	✓	✓	--	91.09%	92.17%	90.36%
✓	✓	✓	✓	92.90%	97.05%	93.14%

## Data Availability

The data that support the findings of this study are available from the corresponding author upon reasonable request.
